# *In silico* determination of novel SARS-CoV-2 envelope protein ion channel inhibitors

**DOI:** 10.1016/j.csbj.2025.06.036

**Published:** 2025-06-26

**Authors:** Nina Kobe, Lennart Dreisewerd, Matic Pavlin, Polona Kogovšek, Črtomir Podlipnik, Uroš Grošelj, Miha Lukšič

**Affiliations:** a National Institute of Biology, Department of Biotechnology and Systems Biology, Večna pot 121, Ljubljana, SI-1000, Slovenia; b Jožef Stefan International Postgraduate School, Jamova cesta 39, Ljubljana, SI-1000, Slovenia; c University of Ljubljana, Faculty of Chemistry and Chemical Technology, Večna pot 113, Ljubljana, SI-1000, Slovenia; d National Institute of Chemistry, Department of Catalysis and Chemical Reaction Engineering, Hajdrihova 19, Ljubljana, SI-1000, Slovenia; e Faculty of Polymer Technology, Ozare 19, Slovenj Gradec, SI-2380, Slovenia

**Keywords:** SARS-CoV-2 envelope protein, Viroporin, Ion channel, Channel blockers, Molecular dynamics simulations, Free energy calculations, Drug discovery

## Abstract

The SARS-CoV-2 envelope protein (2-E^PRO^), a viroporin crucial for viral pathogenesis, is a promising target for antiviral drug development as it is highly conserved and functionally important. Although it is a promising therapeutic target for the treatment of COVID-19, it has often been overlooked in previous studies. In this study, a high-throughput virtual screening of nearly one billion compounds was performed, followed by rigorous filtering and re-docking. Eight best-scoring and chemically versatile lead candidates were identified. In molecular dynamics simulations, three of these ligands showed stable protein-ligand complexes occupying the 2-E^PRO^ channel pore. Among these, ZINC001799167680 (L3) and ZINC001081252239 (L2) exhibited the strongest binding affinity, with key interactions at residues ASN15, THR11 and GLU8 identified by Molecular Mechanics Poisson-Boltzmann Surface Area analysis. All ligands were compared with the known inhibitor rimantadine and showed stronger binding to the protein. These *in silico* results highlight the potential of focusing on the 2-E^PRO^ ion channel in the development of novel COVID-19 therapeutics and pave the way for further *in vitro* and *in vivo* studies.

## Graphical abstract

**Figure:**
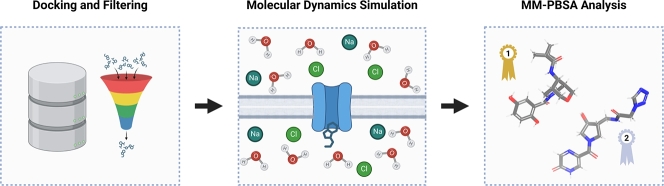

## 1 Introduction

Although vaccination is an effective preventive measure, individuals with a generally weak immune response and groups that cannot be vaccinated, such as cancer patients, remain at risk of severe complications from a SARS-CoV-2 infection [1]. Monoclonal antibodies can partially compensate for these shortcomings, but suffer from high production costs, limited shelf life, strict storage requirements and the need for administration by medical personnel, which limits their overall availability [2], [3]. In addition, other currently available COVID-19 therapeutics offer only partial benefits to patients. Dexamethasone, a glucocorticoid targeting the immune system, only moderately reduces mortality in patients suffering from COVID-19-induced respiratory decompensation [4]. Remdesivir, an RNA polymerase inhibitor of the host cell, blocking the replication cycle of the virus, also has no beneficial effect for severely affected patients [5], [6]. Finally, the antiviral drug ritonavir-boosted nirmatrelvir, which targets the major protein (M^PRO^), only provides satisfactory protection in adults with mild or moderate disease within five days of symptom onset [7]. Taking this into account illustrates the need for a further extension of the repertoire of readily available and effective antiviral drugs. In this case, a drug that specifically targets a conserved site in the viral machinery and thus reduces the risk of mutation-induced affinity losses, is desirable. In addition, the drug target should play a pivotal role in viral pathogenesis and ensure a significant reduction in cellular damage and viral replication.

**Fig. 1:**
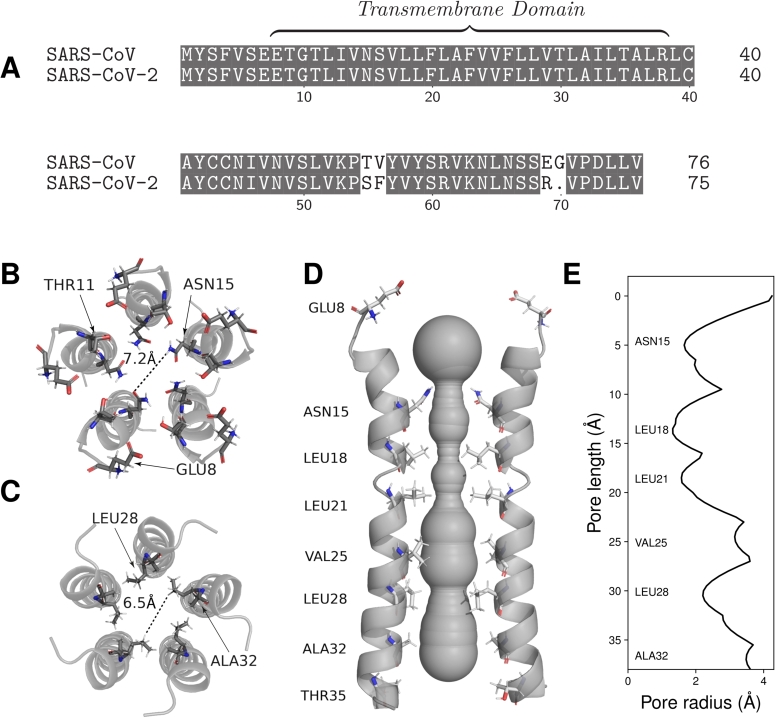
**A** Sequence alignment of the SARS-CoV and SARS-CoV-2 E^PRO^, respectively. Identical amino acids are highlighted in grey. **B** Transversal view onto the N-termini of the transmembrane domain of the 2-E^PRO^ ion channel (PDB ID 7k3g) [8]. For each subunit, the amino acid residues of GLU8, THR11 and ASN15 are displayed, representing a relevant region for inhibitor binding and ion selectivity of the N-terminal vestibule [8]. **C** Transversal view onto the C-termini of the 2-E^PRO^ ion channel. For each subunit, LEU28 and ALA32 residues are displayed, which are inward-facing and channel-constricting amino residues. **D** The Caver 3.0 [9] calculated volume of the ion channel, which extends along the longitudinal axis of the five 2-E^PRO^ subunits. **E** The pore radii along the longitudinal axis of the calculated volume of the ion channel.

The SARS-CoV-2 envelope protein (2-E^PRO^), a structural protein and the smallest (8-12 kDa) of the major proteins of SARS-CoV-2 [10], is a promising candidate with regard to the criteria of genetic conservation and patho-mechanistic relevance. However, due to a lack of research, current knowledge on 2-E^PRO^ is mainly limited to studies on envelope proteins (E^PRO^) of other betacoronaviruses (*β*-CoVs). One example is SARS-CoV, which shares 80% genetic homology with SARS-CoV-2 [11]. It is noteworthy that its envelope protein (1-E^PRO^) has only minor deviations in the amino acid sequence of 2-E^PRO^ (Fig. 1A). Murine hepatitis virus (MHV), a surrogate virus of SARS-CoV and SARS-CoV-2 [12], is frequently used to study the pathogenesis and immune responses of *β*-CoVs [13]. One of these studies has shown that despite being highly expressed in host cells, only a small fraction of E^PRO^ is integrated into the viral envelope [14]. There, it is thought to interact with other structural proteins that provide structural support and mediate release [15]. For SARS-CoV 1-E^PRO^, it was found that points of concentration are located at the endoplasmic reticulum (ER), the Golgi apparatus and the ER-Golgi intermediate compartment (ERGIC) [16]. All three regions are involved in the budding and assembly of coronaviruses [17]. Further, it was found that E^PRO^ monomers oligomerise into homopentameric, ion-conducting viroporins [10], [16], [18]. These cation-selective channels can contribute significantly to the regulation of ion balance and the creation of microenvironments in host cells [14]. This is supported by the fact that recombinant Corona-viruses lacking E^PRO^ show a greatly reduced viral titer and lower maturity or are completely deficient in competent offspring [19], [20]. Inhibition of 2-E^PRO^ in transgenic mice infected with SARS-CoV-2 resulted in a significant reduction in viral load and cytokine inflammatory marker levels in the lung, demonstrating the complex role of 2-E^PRO^ in viral replication and cellular damage [10]. These processes contribute to clinical manifestations such as cytokine storms, which can significantly worsen disease progression and patient outcomes [10], [21], [22]. Other SARS-CoV-2 symptoms, extending beyond respiratory issues, such as neurological manifestations, have been linked to 2-E^PRO^ expression, promoting proliferation and inflammation in glioblastoma cells [23]. In addition, 2-E^PRO^ has been shown to strongly upregulate tumour necrosis factor (TNF) production, a key cytokine predictive of COVID-19 severity and mortality [24]. Overexpression of TNF has been associated with taste disorders [25], suggesting that 2-E^PRO^ may be a major contributing factor. Furthermore, 2-E^PRO^ expression was found to play a crucial role in regulating metabolic activity and inducing ER stress by altering ER Ca^2+^ homeostasis, thereby affecting cellular repair mechanisms and homeostasis of most human tissues and organs [26].

Several *in silico* and *in vitro* based drug-screening and NMR studies have identified a relevant ligand binding region inside the alpha-helical pentameric channel, providing a viable target for cation flow obstruction [8], [27], [28], [29], [30]. This region can be roughly localised at protein residues 15-32, which represent a hydrophobic and in-diameter constricted stretch of the channel (Fig. 1B-E). A small set of ligands such as proanthocyanidins [28], amantadine (AMT), rimantadine (RMT) and 5-(*N,N*-hexamethylene)amiloride (HMA) [8], [31] were found to bind at or near this region, partly with experimentally confirmed beneficial effects for infected cells via antiviral efficiency tests, SPR assays [28] and patch clamp electrophysiology measurements [18], [31].

**Fig. 2:**
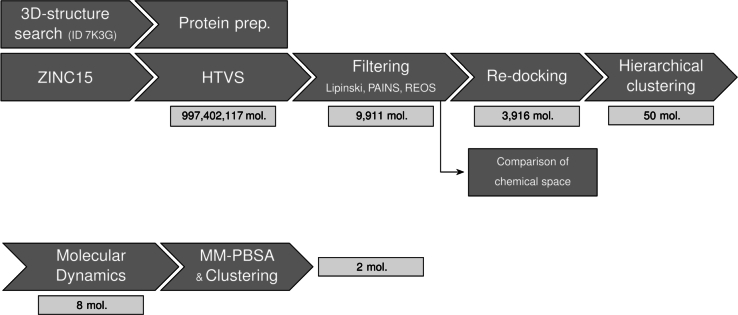
The workflow for screening 2-E^PRO^ inhibitors: HTVS docking (∼10^9^ molecules), filtering, re-docking and compound prioritisation (50 molecules). For eight structurally diverse ligands with high docking scores, all-atom molecular dynamics simulations were performed, and trajectories were analysed using MM-PBSA. The number of molecules (mol.) used at the beginning of each step is indicated below each step.

In this work, we initially performed a high-throughput virtual screening (HTVS) of potential channel inhibitors of the 2-E^PRO^ ion channel using a large set ($∼10^{9}$) of compounds from the ZINC15 database [32]. Post-docking, we applied selective filtering methods to select molecules with high druglikeness and a lower risk of undesired off-target effects. The filtered chemical space was visualised and evaluated via a novel clustering protocol that involved two dimensionality reduction techniques: principal component analysis (PCA) and Pairwise Controlled Manifold Approximation (PaCMAP), the latter being a state-of-the-art algorithm [33]. By using a more computationally intensive and accurate re-docking step, additional chemoinformatics tools and all-atom explicit solvent molecular dynamics (MD) simulations, we prioritised the most promising candidates. From almost a billion molecules in the database, we have identified two ligands that show promising binding affinities *in silico*. The workflow of the present study, highlighting the crucial steps and the reduction in the number of ligand candidates, is shown in Fig. 2.

## 2 Materials and methods

### 2.1 Molecular docking workflow

The entire ZINC15 database [32] was downloaded, yielding a total of 997,402,117 molecules. The 2-E^PRO^ NMR structure of Mandala et al. (PDB ID 7K3G) with a resolution of 2.1 Å was selected [8]. The Schrödinger Protein Preparation Wizard [34], [35] was used to preprocess the protein structure. A restrained minimisation of the structure was performed, followed by an optimisation of the protein's hydrogen bond network, after which a receptor grid was generated. Subsequently, the entire database was docked [36], [37] into the prepared receptor binding site, and 0.001‰ of top-scoring compounds were considered in the next step. The re-dock step was conducted with Schroedinger software involving an HTVS, a standard and an extra-precision step via *Glide*, using standard settings [38]. Further details on the protocol of the docking steps can be found in the Supplementary Information (SI) file (Section S1).

### 2.2 Chemical space investigation and filtering

After docking, six molecular descriptors were generated for each molecule using RDKit software (see Table S1 in the Supplementary Information (SI) file) [39]. In order to discriminate for molecules with higher druglikeness and oral bioavailability, a strict (so-called Lipinski) filter–tolerating one violation per molecule–was applied [40], [41]. Specifically, compounds were excluded if they exceeded two or more of the following thresholds: molecular weight >500 g/mol, logP >5, more than 5 hydrogen bond donors, more than 10 hydrogen bond acceptors, more than 10 rotatable bonds, or a topological polar surface area (TPSA) above 140 Å^2^. This step was followed by the detection and exclusion of motifs resembling pan-assay interference compounds (PAINS) [42] and moieties that could be toxic or reactive (REOS) [43].

To gain further insight into how the chemical space of the filtered dataset is composed, MACCS keys [44] were generated via RDKit software and low variance features were removed from the dataset. In contrast to path-based fingerprints, MACCS keys are structural keys that encode the presence of specific functional groups for an organic molecule in a binary vector. Optimised for bioactive substances, MACCS keys provide a global overview of the biologically relevant functional group space and allow discrimination between molecules based on their predominant functional groups. Two dimensionality reduction techniques–PCA and PaCMAP–were used to group the molecules using the generated structural keys. The retained principal components were submitted to the PaCMAP algorithm using default parameters after parameter optimisation (see Fig. S2 in the SI file). The Density Based Spatial Clustering of Applications with Noise (DBSCAN) algorithm [45] was employed for classifying the clustered data, using a local radius for expanding clusters of 0.44 and at least 4 neighbours. After docking, the MACCS keys were generated as described above. The pairwise distances were calculated with the aid of SciPy software [45] via scipy.spatial.distance.pdist by employing the Euclidean distance as the distance metric. In the following, hierarchical clustering was performed using the linkage function of the scipy.cluster.hierarchy module using the Ward variance minimisation algorithm [46] to group similar molecules. After clustering, eight lead candidates were selected based on the criteria of docking score and structural diversity. Details on chemical space monitoring are given in the SI file (Section S2).

The protein-ligand interactions were visualised using PyMol [47] and BioRender software and the volume of the 2-E^PRO^ channel was calculated using Caver 3.0 software [9].

### 2.3 Molecular dynamics simulations and MM-PBSA analysis

The ligand-protein complexes for the eight candidates selected in the previous step and RMT were used as inputs for classical all-atom molecular dynamics (MD) simulations. Each complex was embedded in a 1-palmitoyl-2-oleoylphosphatidylcholine (POPC) lipid bilayer (the total number of lipid molecules in both layers was 778) located in a rectangular simulation box (edge lengths of the box 165 x 165 x 97 Å). The assembled ligand-protein-lipid systems were solvated with ∼46,490 TIP3P water molecules. 117 Na^+^ and 117 Cl^−^ ions were added to mimic a physiological 0.150 mol/L NaCl solution. The complexes were embedded in the membrane, and the system was solvated using the CHARMM-GUI web server [48], [49], [50], [51], [52], [53], [54], [55]. Each system consisted of a total of ∼325,000 atoms. GROMACS software [56] was used to perform the molecular dynamics simulations of the equilibrated 2-E^PRO^ ligand complexes at 37 ^∘^C and 1 bar. Amber FF19SB was used to describe the protein [57] and the membrane was described with the Lipid21 force field [58]. GAFF2 was used to describe the ligand. The electrostatic parameters of the ligand were generated with the Amber21 module ANTECHAMBER [59], [60].

The protein-ligand binding free energy and the per-residue contributions were calculated via single trajectory Molecular Mechanics Poisson-Boltzmann Surface Area (MM-PBSA) calculations using gmxMMPBSA [61]. Details on the MD protocols and the trajectory analysis can be found in the SI file (Section S3).

## 3 Results

### 3.1 HTVS docking

HTVS docking of nearly one billion compounds from the ZINC15 database resulted in a smaller subset of 9,911 ligand molecules. Of these, 3,916 (43.0%) passed the subsequent filtering step, where a total of 44 Lipinski, 51 PAINS and 5,959 REOS violations were registered (see Fig. S1 in the SI file).

### 3.2 Chemical space visualisation

**Fig. 3:**
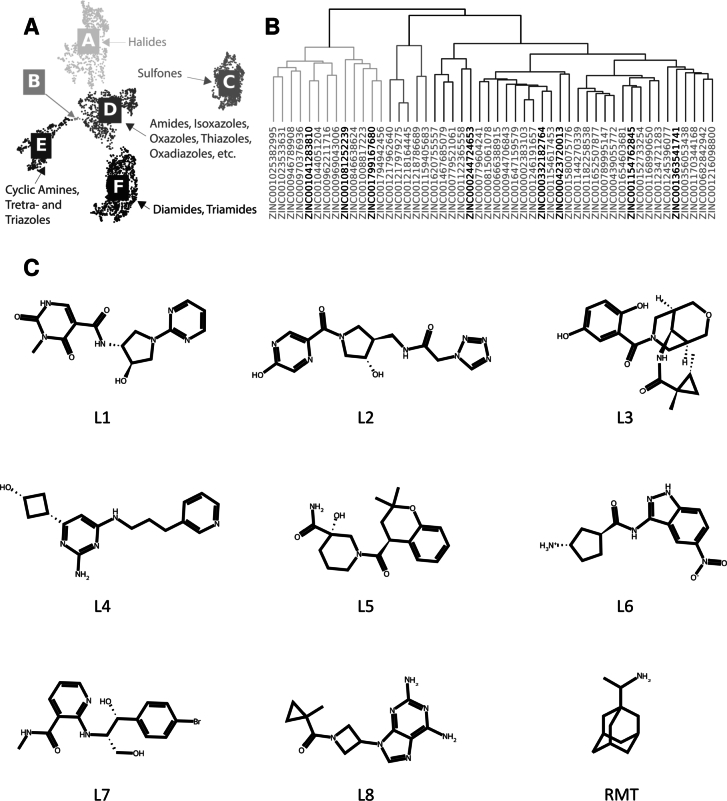
**A** Chemical space of the filtered dataset after dimensionality reduction and clustering, displaying five clusters (A-F). Each cluster represents recurring functional group motifs within the cluster. An interactive plot can be found under: https://sars-cov-2-envelope-protein.gitlab.io/e-protein-data/. **B** Hierarchical clustering of the top 50 scoring compounds of the re-docking step. Prioritised compounds are highlighted in black. **C** Selection of lead compounds (L1-L8) and rimantadine (RMT). The selected candidates L1-L8 correspond to the highlighted compounds in the dendrogram (panel **B**) in descending order from left to right.

To further explore the remaining chemical space, MACCS keys were generated from the filtered dataset and clustering was performed. This process resulted in the formation of six distinct clusters (Fig. 3A), each characterised by recurring functional group motifs. Cluster A consists of molecules containing halide groups, while Cluster B contains mainly of tetrahydrofuran-containing compounds. Cluster C represents molecules with sulfone groups. Cluster D includes compounds featuring functional groups such as amides and five-membered heteroaromatic rings, including isoxazoles, oxazoles and thiazoles. Cluster E consists of nitrogen-containing functional groups, such as cyclic amines, triazoles and tetrazoles, and Cluster F primarily contains molecules bearing diamides and triamides, often in combination with groups such as spiro compounds, ethers, alcohols and amines. An interactive visualisation of the chemical space can be found at https://sars-cov-2-envelope-protein.gitlab.io/e-protein-data/.

### 3.3 Re-docking and ligand prioritisation

**Fig. 4:**
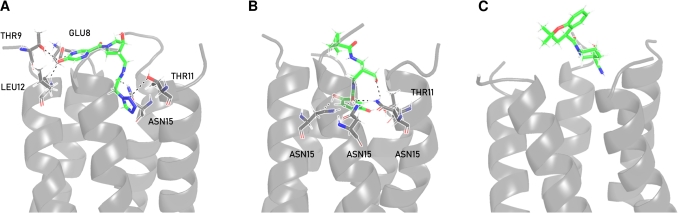
Most representative clusters depicting hydrogen-bond-interactions and binding poses of the final compound selection of L2, L3 and L5 corresponding to **A**-**C**, respectively. Protein moieties are depicted in grey. The ligand is depicted in green. Nitrogen- and oxygen-atoms are depicted in blue and red, respectively. Hydrogen-bonds are shown as dashed black lines.

The remaining 3,916 compounds from the HTVS and filtering steps were re-docked, and the top 50 compounds identified for further evaluation (Fig. 2). Hierarchical clustering was performed for these compounds, resulting in three main branches (Fig. 3B). The leftmost branch contains molecules with bridged bicyclic motifs, *rac*-(3*R*,4*S*)-4-aminopyrrolidin-3-ol moieties and indane derivatives such as indoles. The middle branch contains ellagic acid, salicylfluorone and 5-chloropyrimidine-4,6-diamine motifs. The rightmost branch contains chromane derivatives as well as a large number of heterocycle-substituted indene moieties. Eight structurally diverse ligand candidates (L1–L8) with high docking scores were selected from these clusters for a further MD study. Their structures are shown in Fig. 3C. For all eight compounds, docking analysis located the binding site at the *N*-terminal vestibule (Fig. 4), with hydrogen bond interactions with ASN15 observed throughout. Two additional hydrogen-bonding interactions were observed for L6 with GLU8 and for all compounds (except L4) with THR11.

### 3.4 MD simulations and MM-PBSA analysis

The selected eight candidates were submitted to 200 ns long MD simulations, and the system with RMT was simulated for 125 ns. The root mean square deviations (RMSD) calculated on the C-*α* atoms of 2-E^PRO^ reach a stable state after ∼100 ns, except for L7 where the MD simulation was extended to 300 ns (see Fig. S3 in the SI file). In the case of RMT, the RMSD was already stable after 50 ns, and the simulation time was therefore shortened to 125 ns (Fig. S3). The average plateau RMSD values ranged from 0.48 to 0.58 nm (Fig. S3), reflecting moderate structural flexibility characteristic of proteins with flexible loops and solvent-exposed regions. However, we note that this flexibility does not impair channel integrity nor does it compromise ligand stability. In addition, slight movements of the pentameric domains and ligand-induced conformational changes also contribute to the overall RMSD. Nevertheless, the secondary structure elements of the protein were well conserved throughout the MD simulations. Analysis of the radius of gyration was continuously stable over the simulated time, suggesting a stable assembly of the 2-E^PRO^ ion channel (Fig. S4 in the SI file). Three candidates form stable complexes within the *N*-terminal vestibule of 2-E^PRO^. These include L2, L3 and L5, while others dissociated from the active site during the simulation. The dissociation of certain candidates is mainly attributed to shallow channel binding (Fig. 4), weak hydrogen bonds and limited van der Waals interactions with the amino acid residues (Figs. S5 and S6 in the SI file). The channel is mainly a hydrophobic stretch and only a few amino acid residues are responsible for the ligand-protein interaction (Fig. 1, Table S3 in the SI file). In addition, the channel offers limited space, leading to steric conflicts between the residues and some of the ligands tested. The number of hydrogen bonds varied considerably for each candidate during the simulation (Fig. S5 in the SI file), indicating an overall dynamic binding regime. The highest populated clusters of L2, L3, L5 and RMT represent 90.8, 75.1, 61.0 and 74.4% of the equilibrated trajectory, respectively. Their protein-ligand interactions are described in the following text.

An MM-PBSA analysis was performed to evaluate the binding affinity of each ligand. The total binding free energies are listed in Table 1. Ligand L3 exhibits the strongest binding, followed by ligands L2 and L5. The  in case of RMT was approximately 1.7-fold (L5) to 2.5-fold (L3) smaller than that of our lead compounds. At the amino acid residue level, GLU8, THR11, LEU12 and ASN15 were found to be major contributors to binding for the top three candidates (see Fig. S6 in the SI file for the per-residue contributions). Throughout the simulation, L3 showed significant binding with three ASN15 and one THR11 side chain from adjacent 2-E^PRO^ monomers (Table S3 in the SI file). Among all candidate structures, L3 demonstrated the strongest binding interactions with ASN15 and THR11 residues. Analysis of the highest populated cluster supports these findings (Fig. 4B) displaying a total of five hydrogen bonds with three adjacent ASN15 residues. These interactions involve the ether group of L3. In addition, the 2-formylhydroquinone moiety of L3 participates via one of its hydroxyl groups and its carbonyl group, with the first acting as a single donor and the latter as a triple acceptor for the three adjacent ASN15 residues. Further, the second hydroxyl group of the 2-formylhydroquinone moiety acts as a hydrogen bond donor for a THR11 residue. The 2-formylhydroquinone moiety of L3 penetrates deepest into the channel, while the bridged cycle above exhibits a space-demanding and rigid geometry in which L3 achieves an almost plug-like conformation in the channel.

**Table 1:** Total binding free energies, , of 2-E^PRO^-ligand complexes calculated with MM-PBSA. For candidates that have dissociated from the protein during the MD simulation time,  is not reported.

Candidate	[kcal/mol]
L1	dissociated
L2	−30.1 ± 3.6
L3	−32.3 ± 2.0
L4	dissociated
L5	−21.7 ± 6.1
L6	dissociated
L7	dissociated
L8	dissociated
RMT	−13.2 ± 2.6

L2, the second highest-scoring candidate, exhibits significant binding with an adjacent LEU12 and GLU8 residue (Table S3). Overall, ASN15 and THR11 still contribute to binding (Fig. S6), while their effect is less pronounced than compared to L3. The highest populated cluster (Fig. 4 A) reveals hydrogen bond interactions between one aromatic nitrogen of the pyrazine moiety and a GLU8 residue, along with an additional hydrogen bond and polar interactions involving the same GLU8 and a THR9 and LEU12 from an adjacent monomer, mediated by the hydroxyl group of the pyrazine moiety. On the opposite side, a THR11 residue exhibits a polar interaction with one nitrogen atom of the tetrazole moiety of L2. Additionally, ASN15 from an adjacent monomer acts as a hydrogen bond donor to the carbonyl oxygen of the neighbouring amide group. In total, six hydrogen bonds and polar interactions involving five residues are observed. L2 spans the entire channel lumen, with its tetrazole group extending deepest into the channel.

For L5, the candidate with the lowest binding free energy score, no significant binding above 1.8 kcal/mol to any residue was observed (Table S3). Clustering analysis of the highest populated cluster (Fig. 4C) confirms this observation. Overall, L5 occupies about one-third of the pore, possibly providing enough space for ions to pass through the channel. Its relatively simple architecture and the fact that it does not form a significant binding may indicate that L5 is not a very specific ligand.

For RMT, a known inhibitor of 2-E^PRO^ [31], no significant binding above 1.8 kcal/mol to any residue was observed (Table S3). Clustering analysis of the highest populated cluster (see Fig. S8 in the SI file) confirms this observation, with no hydrogen bonds present. Overall, RMT exhibits a space-demanding and rigid geometry, similarly to L3, effectively occupying space in the channel. RMT's interactions appear to be dominated by its apolar adamantane core, while contacts via its amino group remain transient. We note that although RMT exhibits a weaker  than L5, it appears to occlude the pore more efficiently.

## 4 Discussion

Several inhibitor molecules are known to bind to 2-E^PRO^ and inhibit its channel activity [10], [31]. There is strong evidence that inhibition of 2-E^PRO^ interrupts viral replication and effectively protects against severe pulmonary and systemic damage that results from COVID-19 [10]. However, to the best of our knowledge, none of the known 2-E^PRO^ inhibitors have been proposed for further clinical trials. One reason for this is the limited attention that 2-E^PRO^ has received in the past, leaving researchers with only a handful of compounds. Secondly, many compounds suffer from cytotoxicity issues [31], effectively excluding them from further clinical development. An interesting chemical is amantadine (AMT), a drug approved for the treatment of Parkinson's disease. It was also used to treat influenza A infections for an extended period of time until widespread resistance in viral strains limited its effectiveness. Interestingly, its antiviral effect is based on the inhibition of influenza A matrix protein 2, an ion channel that is relevant for the release of the viral genome into the host cell [62], [63]. Although smaller studies initially suggested that AMT could be a suitable candidate for the treatment of SARS-CoV-2 infections [64], [65], a more recent study found no significant benefit for COVID-19 patients when its effect was compared with that of other antivirals [66].

We therefore firmly believe that more antiviral candidates are needed for the successful development of inhibitors, as complications can quickly lead to the end of *in vitro* and *in vivo* trials. Our proposed ligands are from a set of candidates that we identified when screening the entire ZINC15 database, which at that time included 997,402,117 molecules. We hereby cover a large chemical space that spans multiple classes of small molecules such as metabolites, drugs and natural products [32]. In the subsequent filtering step, we selected for druglike molecules and excluded compounds with known cytotoxic motifs and those that account for promiscuous binding behaviour [40], [41], [42], [43]. We have used a visualisation approach that focuses on common functional group motifs, allowing us to explore the biologically relevant chemical space and monitor the subsequent actions leading to the selection of final candidates. Our filtered molecules were subjected to a re-docking procedure followed by classical MD simulations and MM-PBSA analysis on three selected top-scoring candidates.

Clustering and MM-PBSA analysis of the candidates revealed intermolecular interactions with the residues ASN15, ALA12, THR11 and GLU8 of the 2-E^PRO^ pentameric viroporin. Previous studies support the importance of ASN15, the first pore-facing and channel-restricting amino acid at the *N*-terminus (Fig. 1B). It was found to interact with a number of experimentally confirmed inhibitors such as 5-(N,N-hexamethylene)amiloride (HMA), AMT [8], [18] and procyanidin [28]. Notably, ASN15ALA mutations of 1-E^PRO^ abolished ion conductance [8], [67], [68], highlighting its importance in the ion transport mechanism of the channel. Interactions with THR11 were also observed experimentally for HMA and procyanidin [8], [28], with THR11ALA mutants of 2-E^PRO^ exhibiting significantly reduced viral replication and virulence due to the absence of 2-E^PRO^ ion channel currents [10]. Finally, GLU8, an exposed amino acid at the N-terminus, was found to interact with procyanidin [28]. In addition, the flexible ring formed by the five GLU8 residues is thought to be an important regulatory part of the channel's cation selectivity filter [8]. The MD simulations performed indicate that two of the eight candidates block the 2-E^PRO^ ion channel over an extended period of time by forming hydrogen-bonds with ASN15 and THR11. Binding affinities were calculated for these candidates, with L3 and L2 emerging as the most promising candidates, thereby outperforming our positive control compound rimantadine (RMT) by an approximate factor of two. A recent *in vitro* study compared the activity of classical viroporin inhibitors—AMT, RMT, and HMA—against 2-E^PRO^ via cell viability and electrophysiological assays in HEK293 cells [31]. RMT showed considerably higher potency than AMT (IC=50 (8.9 ± 2.2) μM vs. (89 ± 27) μM), while HMA was the most potent (IC=50 (1.5 ± 0.3) μM). Patch-clamp recordings directly measuring channel activity further demonstrated this potency trend, with IC_50_ values of (3.6 ± 0.6) nM for RMT, (24.1 ± 6.5) nM for AMT, and (1.9 ± 0.3) nM for HMA. However, unlike RMT and AMT, which were not cytotoxic within the tested concentration range, HMA exhibited pronounced cytotoxicity at concentrations above 10 μM, suggesting RMT as a potent and comparatively safe 2-E^PRO^ inhibitor and thus a suitable benchmark compound for assessing the relative performance of the novel candidates L2 and L3.

There are still groups at risk of developing life-threatening COVID-19 complications, such as cancer patients, for whom the Omicron strain was the deadliest in the US compared to all previous SARS-CoV-2 strains [69]. Herein, 2-E^PRO^ is an antiviral target that plays an important role in viral replication and pathogenesis of SARS-CoV-2. The consistency of our study with experimental findings from previous studies emphasises the relevance of our findings and the potential inhibitory properties of our presented lead candidates against 2-E^PRO^. Intriguingly, the high evolutionary conservation of 2-E^PRO^ suggests that antiviral drugs are less prone to suffer from antiviral resistance. Taken together with the suspected presence of other viroporins in SARS-CoV-2 [70] and the frequent occurrence of viroporins in a diverse range of viruses [71], this may point to pathways for the development of antiviral drugs with pan-corona or even broad-spectrum activity. Many pathomechanistic details of SARS-CoV-2 are still being uncovered, such as recent findings suggesting that several variants can form stable complexes with monoamine oxidase enzymes (MAOs) [72], potentially disrupting their function and contributing to oxidative stress and neurodegenerative processes in COVID-19-related neurological complications [73], [74], [75], [76], [77], [78]. Other examples include evidence of 2-E^PRO^ altering ER calcium homeostasis by interfering with sarco/endoplasmic reticulum calcium ATPase function, mimicking host regulins and thereby reducing ER Ca^2+^ reuptake [79]. This illustrates the need for further research to elucidate the full spectrum of virus–host interactions and their implications for long-term disease outcomes as well as for the design of novel therapeutic strategies.

## 5 Conclusion

This study presents a comprehensive computational approach to identify potential inhibitors of 2-E^PRO^, a highly conserved viroporin crucial for viral replication and pathogenesis. The high-throughput virtual screening of a vast chemical space, followed by rigorous filtering and re-docking, led to the identification of eight promising candidates. Molecular dynamics simulations and MM-PBSA analysis showed that among these eight ligands, two proved to be the most promising inhibitors (L2 and L3, *cf*. Fig. 3).

This work highlights the synergy of using advanced chemoinformatics and simulation techniques to target the 2-E^PRO^ ion channel, which has been underexplored despite the important role of 2-E^PRO^ in COVID-19. The results suggest a potential avenue for developing broad-spectrum antiviral therapies targeting viroporins and potentially minimising resistance associated with viral mutations. Future efforts should focus on synthesising the two lead compounds—both L2 and L3 are not commercially available—and on their experimental validation. Validation should include *in vitro* assays to confirm ion-channel inhibition and cytotoxicity assessments, including potential off-target effects such as non-specific binding to host ion channels. Ultimately, *in vivo* testing of lead compounds is essential to evaluate their efficacy and safety. This is a crucial step towards their potential clinical application in the effort to manage COVID-19 and related viral diseases.

## Ethics statement

We declare that no human or animal subject was used in this study. This work was purely computational whereas all procedures were performed in compliance with relevant laws and institutional guidelines in an area of research that does not require institutional approval by an ethical committee.

## Funding sources

The authors acknowledge the financial support from 10.13039/501100004329The Slovenian Research and Innovation Agency (research core funding Nos. P2-0152 (L.D.), P4-0407 (N.K., P.K.), P2-0421 (M.P.), P1-0179 (U.G) and P1-0201 (M.L., Č.P.)). M.L. acknowledges partial support through NIH RM1 award “Solvation modeling for next-gen biomolecule simulations” (grant No. RM1GM135136). The authors also acknowledge the Ažman high-performance computing (HPC) centre at the National Institute of Chemistry and HPC RIVR consortium (HPC Vega) for computing resources and the support of the Centre for Research Infrastructure at the University of Ljubljana, Faculty of Chemistry and Chemical Technology, which is part of the Network of Research and Infrastructural Centres UL (MRIC UL) and is financially supported by the 10.13039/501100004329ARIS (Infrastructure programme No. I0-0022).

## CRediT authorship contribution statement

**Nina Kobe:** Writing – review & editing, Visualization, Validation, Methodology, Investigation, Conceptualization. **Lennart Dreisewerd:** Writing – review & editing, Software, Methodology, Investigation, Conceptualization. **Matic Pavlin:** Writing – review & editing, Software, Methodology, Investigation, Conceptualization. **Polona Kogovšek:** Writing – review & editing, Investigation, Conceptualization. **Črtomir Podlipnik:** Writing – review & editing, Supervision, Software, Methodology, Conceptualization. **Uroš Grošelj:** Writing – review & editing, Resources, Investigation, Conceptualization. **Miha Lukšič:** Writing – review & editing, Visualization, Supervision, Software, Project administration, Methodology, Investigation, Funding acquisition, Formal analysis, Data curation, Conceptualization.

## Declaration of Competing Interest

The authors declare that they have no known competing financial interests or personal relationships that could have appeared to influence the work reported in this paper.

## Data Availability

The original contributions presented in the study are included in the article/supplementary material. Further inquiries can be directed to the corresponding author.

## Appendix A Supplementary material

MMC Supplementary material file contains details about the docking protocol and filtering, the visualisation and monitoring of the chemical space, the molecular dynamics simulations protocol and the Molecular Mechanics/Poisson-Boltzmann analysis. MMC
