# Supplementary material for: In silico determination of novel SARS-CoV-2 envelope protein ion channel inhibitors
Source: Comput Struct Biotechnol J. 2025 Jun 26;27:2823–31. doi: 10.1016/j.csbj.2025.06.036 (PMC12268682; doi:10.1016/j.csbj.2025.06.036)
Supplement: MMC — Supplementary material file contains details about the docking protocol and filtering, the visualisation and monitoring of the chemical space, the molecular dynamics simulations protocol and the Molecular Mechanics/Poisson-Boltzmann analysis. [file mmc1.pdf]

Supplementary Information:

In silico determination of novel SARS-CoV-2 envelope protein ion channel inhibitors

Nina Kobe[1,2], Lennart Dreisewerd[3,4], Matic Pavlin[4,5], Polona Kogovšek[1], Črtomir Podlipnik[3], Uroš Grošelj[3], and Miha Lukšič[3]

[1]National Institute of Biology, Department of Biotechnology and Systems Biology, Večna pot 121, SI-1000, Ljubljana, Slovenia

[2]Jožef Stefan International Postgraduate School, Jamova cesta 39, SI-1000, Ljubljana, Slovenia

[3]University of Ljubljana, Faculty of Chemistry and Chemical Technology, Večna pot 113, SI-1000, Ljubljana, Slovenia

[4]National Institute of Chemistry, Department of Catalysis and Chemical Reaction Engineering, Hajdrihova 19, SI-1000, Ljubljana, Slovenia

[5]Faculty of Polymer Technology, Ozare 19, Slovenj Gradec, SI-2380, Slovenia

S1 Docking Protocol and Filtering

The initial docking campaign was carried out with *CmDock* software [1, 2, 3], which is based on the *rDock* software [4]. Here, flexible ligand docking was performed with 100 iterations per molecule. Otherwise, the default settings of *rDock* were used.

After docking, 9,911 compounds (which accounted for 0.001‰ of the top-scoring compounds) were retained, and six molecular descriptors were generated for each molecule. A strict filter was applied that tolerated one violation per molecule [5, 6]. The employed descriptors and the violation thresholds can be found in Table S1. After this initial step, the molecules were screened for functional group motives that account for cytotoxicity (REOS) [7] and promiscuous binding properties (PAINS) [8]. 3,916 (42.98%) passed the subsequent filtering steps, in which a total of 44 Lipinski, 51 PAINS and 5,959 REOS violations were registered (Figure S1).

Within the re-docking campaign, an HTVS step was followed by a standard and extra-precision step in *Glide*, respectively. The standard settings of the Schroedinger software were used [9].

Table S1: Filtering criteria and used descriptors with the employed violation thresholds.

Criteria	Descriptor	Violation threshold
Molecular weight	`rdkit.Chem.Descriptors.MolWt`	500 g/mol
Octanol-water partition ratio	`rdkit.Chem.Crippen.MolLogP`	5
Hydrogen bond donors	`rdkit.Chem.Lipinski.NumHDonors`	5
Hydrogen bond acceptors	`rdkit.Chem.Lipinski.NumHAcceptors`	10
Number of rotatable bonds	`rdkit.Chem.Lipinski.NumRotatableBonds`	10
Topological polar surface area	`rdkit.Chem.Descriptors.TPSA`	140 $Å^2$

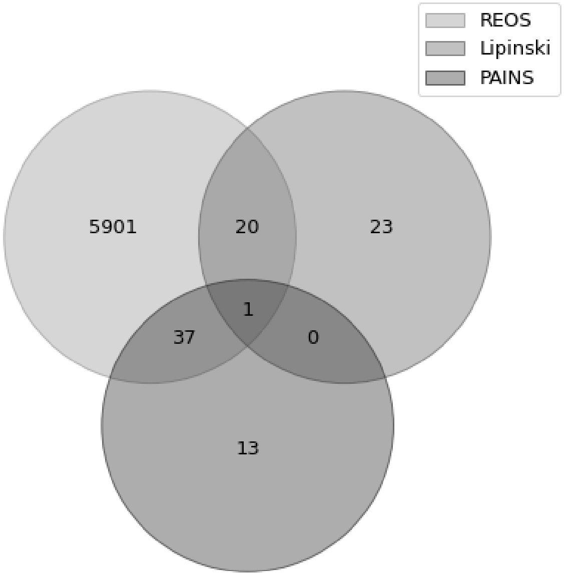

Figure S1: Venn diagram depicting the overlap in violations that were registered for REOS, Lipinski and PAINS filtering of the obtained data from the first docking step.

S2 Visualisation and monitoring of the chemical space

To gain further insight into the chemical space of the filtered dataset, MACCS keys were generated using *RDKit* software [10], and low variance features were removed from the dataset. Two dimensionality reduction techniques were used: principal component analysis (PCA) and Pairwise Controlled Manifold Approximation (PaCMAP) [11]. The first ten principal components, which account for 57% variance, were retained and subjected to the PaCMAP algorithm.

The most important PaCMAP parameters were optimised (Figure S2): `n_neighbors, MN_ratio, FP_ratio`. In addition, `num_iters` has been optimised. It was found that the default values provide the best results. It should be noted that the parameter `init` was excluded from the optimisation, as its influence is estimated to be negligible [11].

The Density Based Spatial Clustering of Applications with Noise (DBSCAN) algorithm [12] was used for clustering the generated data, using $\epsilon = 0.44$ and `min_samples` = 4.

The following populations were found during classification: Cluster A: 290, Cluster B: 9, Cluster C: 805, Cluster D: 708, Cluster E: 485, and Cluster F: 980 (see Figure 3A in the main article). Pre-processing the data using PCA before using the main dimensionality reduction algorithm is a well-established approach, hereby, e.g., suppressing noise and speeding up the computation [13, 14]. In contrast to other dimensionality reduction methods, PaCMAP is robust to pre-processing decisions and achieves fast runtimes even with large data sets [14].

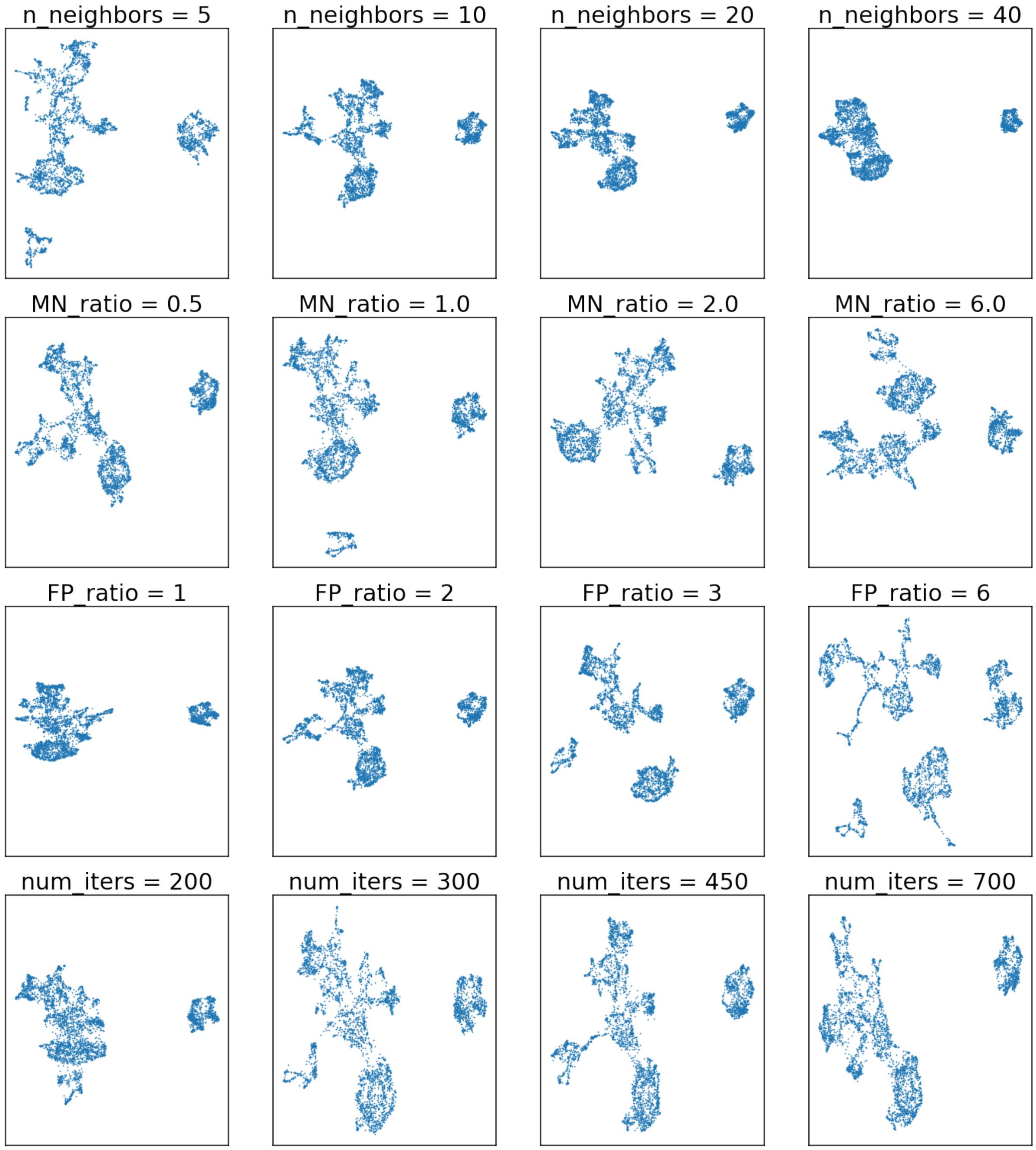

Figure S2: PaCMAP parameter optimisation. From top to bottom: Optimisation of the number of neighbours (default value: `n_neighbors` = 10), optimisation of the ratio of mid-near points (default value: `MN_ratio` = 0.5), optimisation of the ratio of further points (default value: `FP_ratio` = 2) and optimisation of the number of gradient steps (default value: `num_iters` = 450).

S3 Molecular Dynamics Simulations and Molecular Mechanics/Poisson-Boltzmann Analysis

MD simulations were performed with GROMACS (v2022.3) software [15]. The preparation protocol consisted of a restrained minimisation step and five equilibration steps that were designed to gradually relax the system while maintaining its original coordinates. First, steepest descent minimisation was performed with a maximum of 50,000 steps. Positional restraints for backbone, sidechain, lipid as well as dihedral restraints were implemented with a force constant of 4000 kJ mol^{-1} nm^{-2}, 2000 kJ mol^{-1} nm^{-2}, 1000 kJ mol^{-1} nm^{-2} and 1000 kJ mol^{-1} nm^{-2}, respectively. Next, a 1 ns-long simulation in the isothermal-isochoric ensemble (NVT), with the same force constants as in the previous step, was performed. The temperature was set to 310.15 K, ensuring that the POPC membrane was above the main phase transition temperature [16, 17]. Nose-Hoover thermostat [18, 19] with a time constant for coupling equal to 1 ps was employed. The time step for the integration of Newton's equations of motion was 2 fs. Periodic boundary conditions and a Verlet cut-off scheme were used (cut-off distance for the short-range neighbour list was 1.2 nm, van der Waals cut-off was 1.2 nm). Electrostatics were treated within PME (distance for the Coulomb cut-off was 1.2 nm) [20]. Bonds with H-atoms were constrained using LINCS algorithm. The neighbour list was updated with a frequency of 20 steps. After the NVT run, four equilibration simulations in the isothermal-isobaric (NpT) ensemble were performed, where constraints were gradually diminished (durations of each NpT step and values of force constants are given in Table S2). Parrinello-Rahman barostat [21] was employed to ensure constant pressure of 1 bar (time constant for pressure coupling was equal to 2 ps, and compressibility was $4.5 \cdot 10^{-5}$ bar^{-1}).

Table S2: Length of four equilibration MD steps for the NpT ensemble and values of force constants for the backbone, sidechain, lipid and dihedral restraints.

NpT step	time [ns]	$k_{backbone}$ [kJ mol^{-1} nm^{-2}]	$k_{sidechain}$ [kJ mol^{-1} nm^{-2}]	k_{lipid} [kJ mol^{-1} nm^{-2}]	$k_{dihedral}$ [kJ mol^{-1} nm^{-2}]
1	5	4000	2000	1000	1000
2	3	1000	500	400	200
3	3	200	50	40	100
4	3	50	0	0	0

The production run, which followed the equilibration, was performed in the isothermal-isobaric ensemble, simulating the system for 200 ns (and in one case 300 ns). Rimantadine (RMT), a known inhibitor of 2-E^{PRO} was simulated for 125 ns.

Backbone-backbone RMSD and radius of gyration were calculated using built-in GROMACS tools. Within the simulation period, both quantities reached a plateau, upon which the system was considered equilibrated (Figures S3 and S4).

H-bond count was performed using VMD (v1.9.4a51) [22]. A donor-acceptor distance of 3.5 Å and angle cutoff of 30 ° were considered. The time evolution of the number of hydrogen bonds between the protein and the ligand is shown in Figure S5.

To identify the most representative structures, the GROMOS clustering algorithm with a C-α RMSD cut-off of 0.21 nm was employed [23].

Free energy calculations (Molecular mechanics/Poisson-Boltzmann Surface Area) were performed by gmxMMPBSA (v1.6.3) [24]. The MM-PBSA analysis was performed over the last 75 ns of the trajectory of the production run, analyzing every 10th frame (every 0.1 ns). The internal and external dielectric constants were set to 2.0 and 80.0, respectively. A solvent excluded surface was used, and the radius of the solvent probe was 0.14 nm. A per-residue decomposition with 1-4 terms added to

internal potential terms was used in the decomposition scheme, and the decomposition data were collected for residues 8-18. Otherwise, the default settings of gmxMMPBSA were used.

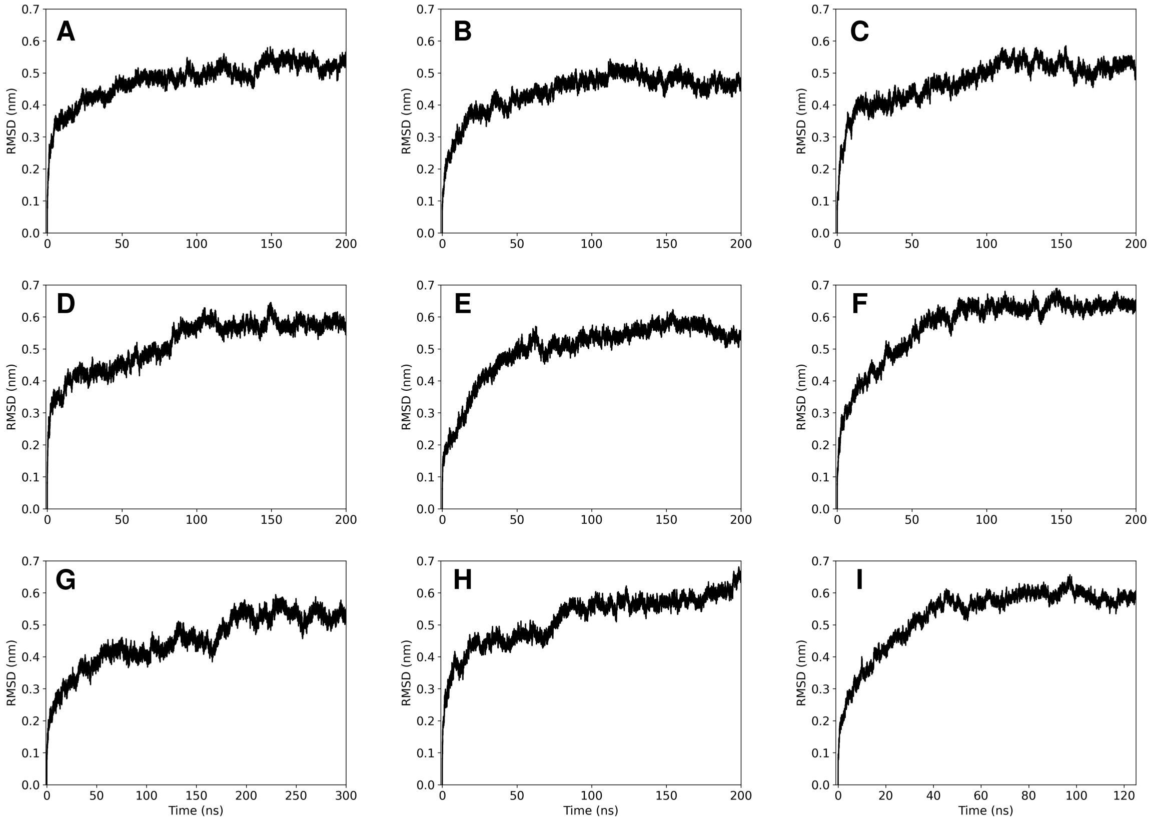

Figure S3: Protein's C-α RMSD as a function of simulation time for the final ligand selection of L1-L8 and RMT corresponding to A-I, respectively.

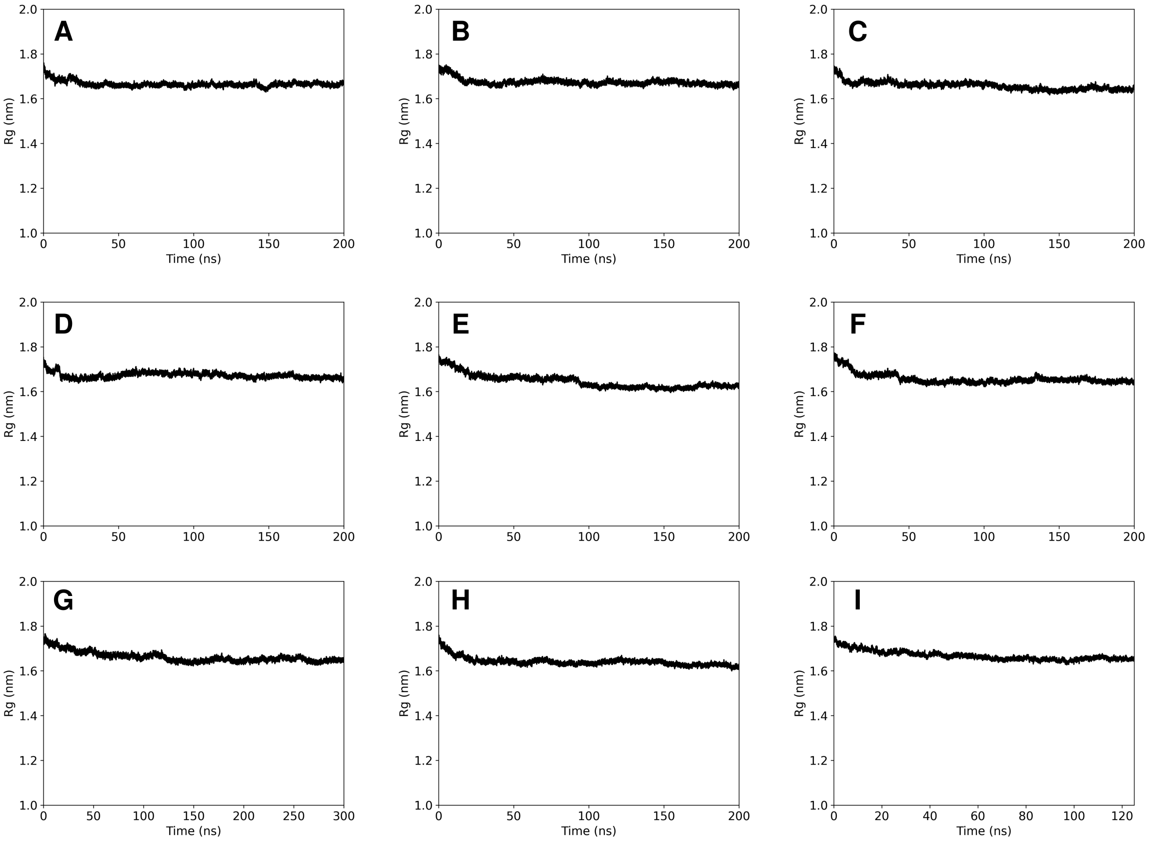

Figure S4: Protein's radius of gyration as a function of simulation time for the final ligand selection. Labels are the same as in Figure S3.

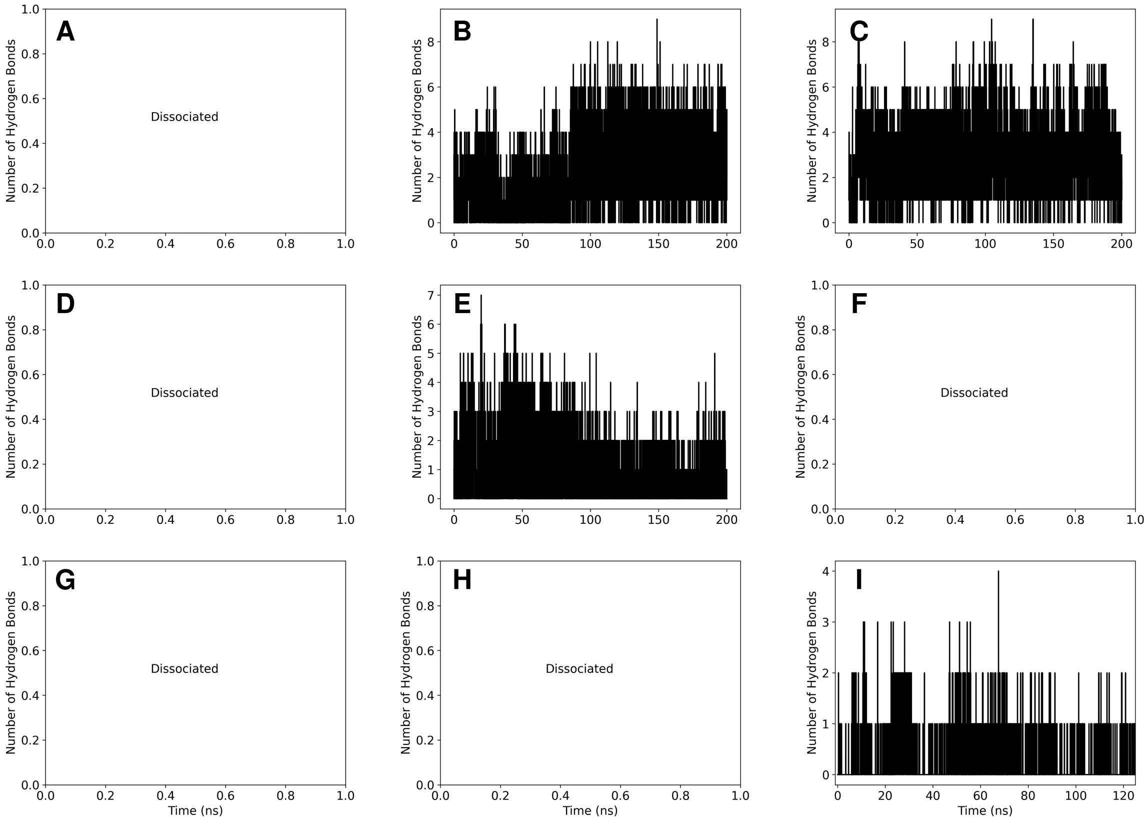

Figure S5: Number of hydrogen bonds formed between the protein and the ligand as a function of simulation time for the final ligand selection. Labels are the same as in Figure S3.

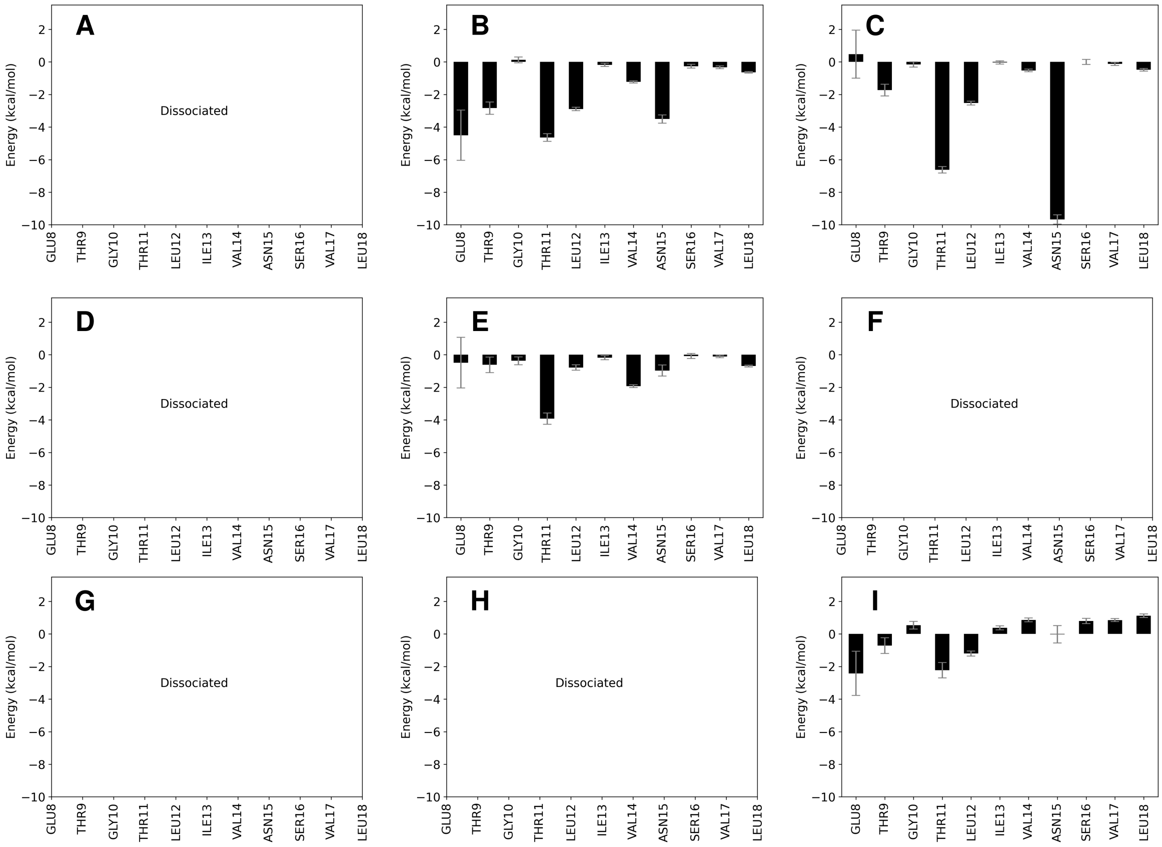

Figure S6: Total MM-PBSA per-residue binding free energy contributions for the final ligand selection. Labels are the same as in Figure S3.

Table S3: MM-PBSA binding free energies of individual protein residues, $\Delta G^{\ominus,\mathrm{res}}_{\mathrm{bind}}$, for which $\Delta G^{\ominus,\mathrm{res}}_{\mathrm{bind}} < -1.80$ kcal/mol. 2-E^{PRO} subunit labels are shown in Figure S7.

Lead	Residue	$\Delta G^{\ominus,\mathrm{res}}_{\mathrm{bind}}$ [kcal/mol]
L2	C:LEU:12	-1.8 ± 0.3
	D:GLU:8	-3.5 ± 0.6
L3	B:THR:11	-2.5 ± 0.4
	C:ASN:15	-2.5 ± 0.3
	D:ASN:15	-2.7 ± 0.3
	E:ASN:15	-3.4 ± 0.4
	C:LEU:12	-2.3 ± 0.5

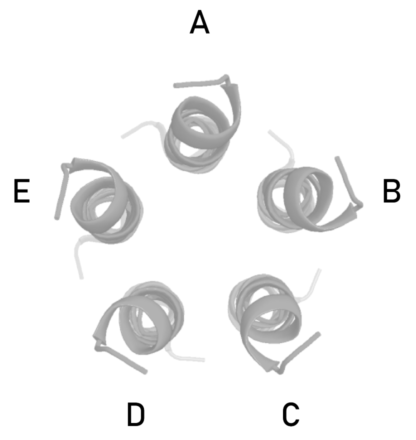

Figure S7: Subunit labels of the homopentameric SARS-CoV-2 Envelope protein.

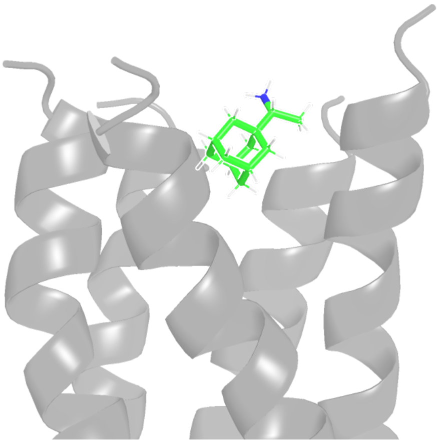

Figure S8: Most representative cluster depicting binding pose of RMT. Protein moieties are depicted in grey. The ligand is depicted in green. Nitrogen-atoms are depicted in blue.

References

[1] Č. Podlipnik, G. Tomšič, M. Jukič, N. Ilc, CmDock: Documentation and repository, https://gitlab.com/Jukic/cmdock, 2022. [Accessed 07-08-2022].

[2] N. Nikitina, M. Manzyuk, M. Jukič, Č. Podlipnik, I. Kurochkin, A. Albertian, Toward crowd-sourced drug discovery: start-up of the volunteer computing project sidock@ home, in: Russian Supercomputing Days, Springer, 2021, pp. 513–524. doi:https://doi.org/10.1007/978-3-030-92864-3_39.

[3] N. Nikitina, M. Manzyuk, Č. Podlipnik, M. Jukič, Performance estimation of a boinc-based desktop grid for large-scale molecular docking, in: International Conference on Parallel Computing Technologies, Springer, 2021, pp. 348–356. doi:https://doi.org/10.1007/978-3-030-86359-3_26.

[4] S. Ruiz-Carmona, D. Alvarez-Garcia, N. Foloppe, A. B. Garmendia-Doval, S. Juhos, P. Schmidtke, X. Barril, R. E. Hubbard, S. D. Morley, rDock: a fast, versatile and open source program for docking ligands to proteins and nucleic acids, PLoS Computational Biology 10 (2014) e1003571.

[5] C. A. Lipinski, F. Lombardo, B. W. Dominy, P. J. Feeney, Experimental and computational approaches to estimate solubility and permeability in drug discovery and development settings, Advanced Drug Delivery Reviews 23 (1997) 3–25.

[6] D. F. Veber, S. R. Johnson, H.-Y. Cheng, B. R. Smith, K. W. Ward, K. D. Kopple, Molecular properties that influence the oral bioavailability of drug candidates, Journal of Medicinal Chemistry 45 (2002) 2615–2623. doi:https://doi.org/10.1021/jm020017n.

[7] W. Walters, M. Namchuk, Designing screens: how to make your hits a hit, Nature Reviews Drug Discovery 2 (2003) 259–266. doi:https://doi.org/10.1038/nrd1063.

[8] J. B. Baell, G. A. Holloway, New substructure filters for removal of pan assay interference compounds (pains) from screening libraries and for their exclusion in bioassays, Journal of medicinal chemistry 53 (2010) 2719–2740. doi:https://doi.org/10.1021/jm901137j.

[9] Schrödinger, LLC, Schrödinger Release 2024-4: Glide, New York, NY, 2024. Software version 2024-4.

[10] G. Landrum, Rdkit: A software suite for cheminformatics, computational chemistry, and predictive modeling, Greg Landrum, 2013.

[11] Y. Wang, H. Huang, C. Rudin, Y. Shaposhnik, Understanding how dimension reduction tools work: An empirical approach to deciphering t-sne, umap, trimap, and pacmap for data visualization, Journal of Machine Learning Research 22 (2021) 1–73. doi:https://doi.org/10.48550/arXiv.2012.04456.

[12] P. Virtanen, R. Gommers, T. E. Oliphant, M. Haberland, T. Reddy, D. Cournapeau, E. Burovski, P. Peterson, W. Weckesser, J. Bright, S. J. Walt, M. Brett, K. M. Joshua Wilson, N. Mayorov, A. R. Nelson, E. Jones, R. Kern, C. Eric Larson, I. Polat, Y. Feng, E. W. Moore, J. VanderPlas, D. Laxalde, J. Perktold, R. Cimrman, E. Ian Henriksen, C. R. Harris, A. M. Archibald, A. H. Ribeiro, F. Pedregosa, Paul van mulbregt, and scipy 1.0 contributors. scipy 1.0: Fundamental algorithms for scientific computing in python, Nature Methods 17 (2020) 261–272. doi:https://doi.org/10.1038/s41592-020-0772-5.

[13] L. Maaten, G. Hinton, Visualizing data using t-sne, Journal of Machine Learning Research 9 (2008).

[14] H. Huang, Y. Wang, C. Rudin, E. Browne, Towards a comprehensive evaluation of dimension reduction methods for transcriptomic data visualization, Communications Biology 5 (2022) 719. doi:https://doi.org/10.1038/s42003-022-03628-x.

[15] M. J. Abraham, T. Murtola, R. Schulz, S. Páll, J. C. Smith, B. Hess, E. Lindahl, GROMACS: High performance molecular simulations through multi-level parallelism from laptops to supercomputers, SoftwareX 1 (2015) 19–25.

[16] S. Leekumjorn, A. K. Sum, Molecular characterization of gel and liquid-crystalline structures of fully hydrated popc and pope bilayers, The Journal of Physical Chemistry B 111 (2007) 6026–6033. doi:10.1021/jp0686339.

[17] C. J. Dickson, R. C. Walker, I. R. Gould, Lipid21: Complex lipid membrane simulations with amber, Journal of Chemical Theory and Computation 18 (2022) 1726–1736. doi:10.1021/acs.jctc.1c01217.

[18] W. G. Hoover, Canonical dynamics: Equilibrium phase-space distributions, Physical Review A 31 (1985) 1695.

[19] S. Nosé, A molecular dynamics method for simulations in the canonical ensemble, Molecular Physics 52 (1984) 255–268.

[20] T. Darden, D. York, L. Pedersen, Particle mesh Ewald: An $N \log(N)$ method for Ewald sums in large systems, The Journal of Chemical Physics 98 (1993) 10089–10092.

[21] M. Parrinello, A. Rahman, Polymorphic transitions in single crystals: A new molecular dynamics method, Journal of Applied Physics 52 (1981) 7182–7190.

[22] W. Humphrey, A. Dalke, K. Schulten, VMD: visual molecular dynamics, Journal of Molecular Graphics 14 (1996) 33–38.

[23] X. Daura, K. Gademann, B. Jaun, D. Seebach, W. F. Van Gunsteren, A. E. Mark, Peptide folding: when simulation meets experiment, Angewandte Chemie International Edition 38 (1999) 236–240.

[24] M. S. Valdés-Tresanco, M. E. Valdés-Tresanco, P. A. Valiente, E. Moreno, gmx_MMPBSA: a new tool to perform end-state free energy calculations with GROMACS, Journal of Chemical Theory and Computation 17 (2021) 6281–6291.
